# Uninterrupted Expression of *CmSIT1* in a Sclerotial Parasite *Coniothyrium minitans* Leads to Reduced Growth and Enhanced Antifungal Ability

**DOI:** 10.3389/fmicb.2017.02208

**Published:** 2017-11-10

**Authors:** Xiping Sun, Ying Zhao, Jichun Jia, Jiatao Xie, Jiasen Cheng, Huiquan Liu, Daohong Jiang, Yanping Fu

**Affiliations:** ^1^State Key Laboratory of Agricultural Microbiology, Huazhong Agricultural University, Wuhan, China; ^2^The Provincial Key Lab of Plant Pathology of Hubei Province, College of Plant Science and Technology, Huazhong Agricultural University, Wuhan, China; ^3^State Key Laboratory of Crop Stress Biology for Arid Areas, College of Plant Protection, Northwest Agriculture and Forestry University, Yangling, China

**Keywords:** *Coniothyrium minitans*, siderophore-mediated iron transport, antifungal substances, *Sclerotinia sclerotiorum*, biological control

## Abstract

*Coniothyrium minitans* is an important mycoparasite of *Sclerotinia sclerotiorum*. In addition, it also produces small amounts of antifungal substances. ZS-1TN1812, an abnormal mutant, was originally screened from a T-DNA insertional library. This mutant showed abnormal growth phenotype and could significantly inhibit the growth of *S. sclerotiorum* when dual-cultured on a PDA plate. When spraying the filtrate of ZS-1TN1812 on the leaves of rapeseed, *S. sclerotiorum* infection was significantly inhibited, suggesting that the antifungal substances produced by this mutant were effective on rapeseed leaves. The thermo-tolerant antifungal substances could specifically suppress the growth of *S. sclerotiorum*, but could not significantly suppress the growth of another fungus, *Colletotrichum higginsianum*. However, *C. higginsianum* was more sensitive to proteinous antibiotics than *S. sclerotiorum*. The T-DNA insertion in ZS-1TN1812 activated the expression of *CmSIT1*, a gene involved in siderophore-mediated iron transport. It was also determined that mutant ZS-1TN1812 produced hypha with high iron levels. In the wild-type strain ZS-1, *CmSIT1* was expressed only when in contact with *S. sclerotiorum*, and consistent overexpression of *CmSIT1* showed similar phenotypes as ZS-1TN1812. Therefore, activated expression of *CmSIT1* leads to the enhanced antifungal ability, and *CmSIT1* is a potential gene for improving the control ability of *C. minitans*.

## Introduction

*Coniothyrium minitans* is an important mycoparasite that can parasitize and destroy both hyphae and sclerotia of fungi of the genus *Sclerotinia. C. minitans* can significantly reduce sclerotial inoculums and inhibit the production of apothecia (Whipps and Gerlagh, 1992; Gerlagh et al., 1999; Diamantopoulou et al., 2000; Bennett et al., 2006; Li et al., 2006; Whipps et al., 2008). *C. minitans* has been used to control Sclerotinia diseases of many vegetable crops (Budge and Whipps, 2001; Jones and Whipps, 2002; Jones et al., 2004; Partridge et al., 2006; McQuilken and Chalton, 2009). *C. minitans* has been registered as the commercial biological control agent, Contans® WG, which is used to reduce and control the diseases caused by *S. sclerotiorum* and *S. minor*. Recently, *C. minitans* was also developed as a biocontrol agent for stem rot of rapeseed (*Brassica napus*) caused by *S. sclerotiorum*, and a provisional registration was issued by the Chinese Ministry of Agriculture in 2015.

In addition to being a mycoparasite, *C. minitans* also produces antifungal and antibacterial substances. However, its ability to produce antifungal substances is very weak. The antifungal substances are usually not observed when dual-culturing with *S. sclerotiorum* on PDA medium and their production can be affected by nutrient factors (McQuilken et al., 2002; Yang et al., 2008). Recent research showed that the production of antifungal substances and mycoparasitism of *C. minitans* are regulated by the ambient pH value (Tomprefa et al., 2011). Under low pH conditions, *C. minitans* can produce more antifungal substances, but when pH values rise, *C. minitans* may produce more fungal cell wall-degrading enzymes to parasitize its hosts (Zeng et al., 2014; Lou et al., 2015). These studies suggest that *C. minitans* has the potential to produce antifungal substances, but the regulation of this production is complicated.

Fe element is an important limiting nutrient for maintaining iron homeostasis. Fungi can uptake iron via siderophores, iron permease, and non-specific divalent metal ion transporters (Van der Helm and Winkelmann, 1994; Haas et al., 2008). Fungal iron metabolism has been studied in detail in the fungal model prototype *Saccharomyces cerevisiae* (Kosman, 2003; Kaplan et al., 2006; Labbéet al., 2007; Winkelmann, 2007). In filamentous fungi, such as *Aspergillus* spp., the biosynthesis, regulation, and physiological function of siderophores have also been studied. The saprobe *A. nidulans* and the human pathogen *A. fumigates* secrete the same siderophores for iron acquisition and possess intracellular siderophores for iron storage (Eisendle et al., 2006; Seifert et al., 2008; Haas, 2014). Siderophores play a crucial role in the pathogenicity of both plant and animal pathogens (Mei et al., 1993; Heymann et al., 2002; Schrettl et al., 2004; Hissen et al., 2005; Oide et al., 2006; Haas et al., 2008; Nevitt and Thiele, 2011; Chen et al., 2013; Moore, 2013; Condon et al., 2014; Ding et al., 2014; Schwartze et al., 2014; Giuliano Garisto Donzelli et al., 2015). The iron-bound siderophores are transported into fungal cells via siderophore iron transporters (SIT), which belong to the major facilitator superfamily (MFS) (Haas et al., 2008).

Previously, we constructed a T-DNA insertional library for *C. minitans*. One of the mutants in this library, ZS-1TN1812, showed an abnormal growth phenotype and could significantly inhibit the growth of *S. sclerotiorum* when dual-cultured on a PDA plate. We further found that the T-DNA insertion likely led to the expression of a gene involved in siderophore-mediated iron transportation. In this study, the potential function of this gene was examined.

## Materials and methods

### Fungal and bacterial strains, plasmids, and culture conditions

The wild-type strain of *Coniothyrium minitans*, ZS-1 (CCAM 041057), which produces pycnidia and conidia on potato dextrose agar (PDA) dishes and abundant conidia in liquid shake culture (Cheng et al., 2003), was used to construct the T-DNA insertional library. ZS-1TN1812, an abnormal mutant, was originally screened from the library. *Sclerotinia sclerotiorum* strain Ep-1PNA367, which was derived from the hypovirulent strain Ep-1PN by single-ascospore-isolation (Xie et al., 2006), was used to examine the antifungal ability of *C. minitans* mutants. *Colletotrichum higginsianum* strain Ch-1 (IMI349061) was donated by Dr Yangdou Wei (University of Saskatchewan). All strains were cultured on PDA at 20–22°C. *Agrobacterium tumefaciens* strain EHA105 was used to transform the wild-type strain ZS-1 as described by Li et al. (2005) and Gong et al. (2007). *Escherichia coli* strain JM109 was used to construct transformation vectors (plasmids) and propagate the plasmids (Gong et al., 2007). The wild-type strain, ZS-1, its mutants, Ch-1, and *S. sclerotiorum* strain Ep-1PNA367 were cultured at 20°C on PDA or PDB, and maintained as PDA slants. *A. tumefaciens* and *E. coli* were cultured in LB medium at 28 and 37°C, respectively, and maintained in LB containing 15–20% glycerol at −20°C.

### Biological characterization of ZS-1TN1812

The growth rate, colony morphology, conidial production, and parasitic ability of ZS-1TN1812 were determined using the methods described by Qin et al. (2011). The mutant was allowed to grow on PDA plates to determine the growth rate and conidial production. To determine the colony morphology, a light microscope was used to observe the hyphal tips. Sclerotia of *S. sclerotiorum* were used to examine the parasitic ability of the mutant.

To determine if ZS-1TN1812 could produce antifungal substances, the mutant was dual-cultured with *S. sclerotiorum* strain Ep-1PNA367 on PDA. ZS-1TN1812 was activated on PDA plates, hyphal agar discs were punched out with a hole punch, and transferred on to fresh PDA plates. The inoculated plates were incubated at 20°C for 4 days and then hyphal agar discs were punched from the activated colonies of *S. sclerotiorum* strain Ep-1PNA367 and were placed at the opposite side of ZS-1TN1812-inoculated plates. The co-inoculated plates were further incubated at 20°C for 7 days. As a control, the wild-type strain ZS-1 was dual-cultured with Ep-1PNA367. This experiment was repeated more than five times.

The antifungal ability of mutant ZS-1TN1812 was further examined on rapeseed leaves. Mutant ZS-1TN1812 was cultured in 100 mL PDB in a 250 mL flask, shaking at 150 rpm at 20°C for 12 days. The hyphae were removed by passing through three layers of filter paper and possible hyphal debris was removed by passing through a bacterial filter (0.22 μm). The filtrate was collected and stored at 4°C before use. Rapeseed leaves were sprayed with the filtrate until fully wet. After drying out at room temperature, the leaves were inoculated with activating hyphal agar discs of *S. sclerotiorum* strain Ep-1PNA367. The inoculated leaves were then placed in a tray and maintained at 100% humidity at room temperature for 1 to 3 days. The lesion diameters induced by strain Ep-1PNA367 were measured with a ruler. The filtrate from the wild-type strain ZS-1 was used instead of the mutant strain ZS-1TN1812, as controls. This experiment was repeated three times.

### Primary characterization of antifungal substances produced by ZS-1TN1812

To characterize the antifungal substances produced by ZS-1TN1812, filtrate was either incubated in a water bath at 60, 80, 100, and 120°C for 30 min or treated with proteinase K (0.05 mg/mL) at 37°C for 2 h following the method described by Jin et al. (1996). The proteinase K digestion was stopped by adding 0.5 μM phenylmethanesulfonyl fluoride (PMSF). To detect the antifungal activity of the treated filtrate, 10% (v/v) of filtrate was amended in 18 mL PDA in a Petri dish (Φ = 90 mm) and activating hyphal agar of *S. sclerotiorum* was inoculated at the center of the plate. The plates were placed at 20°C for 2 days. The non-treated filtrate and PDA, without any emendation, were used as controls. Furthermore, *C. higginsianum* was used instead of *S. sclerotiorum* to repeat this experiment, but the plates were incubated at 20°C for 108 h. This experiment was repeated four times.

### DNA extraction and southern blot analysis

The mycelia mass of the mutants and wild-type strain ZS-1 were harvested 4 days after growing on cellophane membrane laid on PDA (CM-PDA, pH 6.2–6.5) plates, and were used for genomic DNA extraction. Genomic DNA extraction was performed with the CTAB method (Sambrook and Russell, 2001). For Southern blot analysis of the T-DNA insertion in ZS-1TN1812 and copy number determination of *CmSIT1* in *C. minitans*, the protocols were performed according to Gong et al. (2007) with minor modifications. HPH and *CmSIT1* fragment were used as probes amplified with primer pairs HPH-SP/HPH-AP and N1812LBFP1(EX)/ N1812LBFP2(EX) respectively.

### Cloning and sequence analysis of the gene with the T-DNA insertion

The sequences flanking the insertional T-DNA were acquired using the inverse polymerase chain reaction (iPCR) technique following the methods described by Meng et al. (2007). Genomic DNA of ZS-1TN1812 was extracted. The primer pairs LB1/Pttrpc01 and LB3/Pttrpc01 are listed in Table 1. The PCR product was ligated to the TA cloning vector, pMD18-T, and then transformed into *E. coli* JM109 cells. The positive clones were sequenced at Beijing Sunbiotech Co., Ltd, and sequences were analyzed with DNAMAN version 5.2.9 software (Lynnon Biosoft). The DNA sequence was used to design additional specific primers N1812SalIL1/N1812LB1, N1812SalIL2/N1812LB2, N1812HindIIIR1/N1812RB1, and N1812HindIIIR2 /N1812RB2 (see Table 1) for inverse PCR amplification to obtain additional flanking genomic DNA. The DNA sequences obtained were used to search a local *C. minitans* genome database with the BLASTn program and then the T-DNA insertion in ZS-1NT1812 was localized and confirmed. Based on the BLASTn result, the T-DNA was inserted at the promoter region of a gene named *CmSIT1*. The full-length cDNA of *CmSIT1* was obtained by RT-PCR amplification.

**Table 1:** The primers used of PCR.

**Primers**	**Sequence (5′-3′)**	**Usage**
Pttrpc01	ATGTCCTCGTTCCTGTCTGCTAATA	First PCR reaction for T-DNA left border
LB-1	AGGGTTCCTATAGGGTTTCGCTCAG	
Pttrpc01	ATGTCCTCGTTCCTGTCTGCTAATA	Second PCR reaction for T-DNA left border
LB-3	GAATTAATTCGGCGTTAATTCAGT	
N1812SalI L1	AGTCGCCAACAACAGGAT	First PCR reaction for gene left border
N1812 LB1	CCATCATTTGGGCTGTAAG	
N1812SalI L2	AGTCGCCAACAACAGGAT	Second PCR reaction for gene left border
N1812 LB2	ACTGAGGGTTGTCGTGTTC	
N1812HindIIIR1	GCTTGTAAACCTGGACCCT	First PCR reaction for gene right border
N1812RB1	GAAGTGGAGTAAACGACCTG	
N1812HindIIIR2	GCTTGTAAACCTGGACCCT	Second PCR reaction for gene right border
N1812RB2	CTTTAGGCAAGCCCACAT	
HPH-SP	TTCTGCGGGCGATTTGTG	The probe for T-DNA copy number
HPH-AP	AGCGTCTCCGACCTGATG	
N1812LBFP1(EX)	TAAGTAGAACACGACAACCCTC	The probe for *CmSIT1* gene copy number
N1812LBFP2(EX)	TGCCTAAAGTAACGCAGATT	
SIT1SP(EX)	CCACTTCCAACCCGACAC	*CmSIT1* primers for RT- PCR
SIT1AP(EX)	CTTACGCCTCCGACAAAT	
PEX14-3′RACE2	CGCCCTCGGTGAAATGGA	*CmPEX14* primers for RT- PCR
P-AP(EX)	AAGAGCCTTGGGAATGAGAT	
Actin-FP	ACCGTGAGAAGATGACCC	*Actin* primers for RT- PCR
Actin-RP	AAGGACAGAAGGCTGGAAG	
qRT-PCR-FP1	TCCTGTTGTTGGCGACTC	*CmSIT1* primers for qRT-PCR
qRT-PCR-RP1	CGGCAGCGACAAGAGTAG	
CmACT289	GTCCGTGACATCAAGGAGAAGC	Actin primers for qRT-PCR
CmACT419	TT GCCAATGGTGATGACCTGAC	
SIT1-SP(OVER-T)	GGATCCCAATGACGGACACCGAAA	*CmSIT1* cDNA
SIT1-AP(OVER-T)	ATCGATGCTTGTAAACCTGGACCCT	

To investigate the possible functions of CmSIT1, the full length of the putative protein was used to search the GenBank database using the BLAST program of the NCBI website. Multiple alignments of CmSIT1 amino acids were conducted using the Clustal X version 2.0 program (Larkin et al., 2007). The 3D structure of CmSIT1 was predicted using the Phyre2 server (Kelley and Sternberg, 2009; Kelley et al., 2015) and RaptorX web server (Källberg et al., 2012).

### RT-PCR for detecting *CmSIT1* and *CmPEX14* expression pattern

To understand the expression pattern of *CmSIT1*, the total RNA was extracted from the mycelial mass, which was harvested after incubating on PDA for 48, 72, 96, or 120 h. The extraction of total RNA was conducted using TRIzol®130 Plus RNA Purification Kit (Invitrogen, USA) and potential DNA contamination was removed by RNase-free DNase I treatment (TaKaRa, Dalian, China), according to the manufacturer's instructions. First-strand cDNA was synthesized using RevertAid™ First Strand cDNA Synthesis Kit (MBI, Fermentas, USA), following the manufacturer's instructions. Total cDNA abundance in the samples was normalized using the *actin* gene as a control, which was amplified by primer pairs Actin-FP/Actin-RP. Primers pairs SIT1SP (EX)/ SIT1AP (EX) and PEX14-3′RACE2/P-AP (EX) for RT-PCR amplification (Table 1).

### Construction of *CmSIT1* overexpression vectors and ZS-1 transformation

Since the expression of *CmSIT1* in the wild-type strain ZS-1 was undetectable with RT-PCR when growing on PDA and the T-DNA insertion led to high expression of *CmSIT1*, we suspected that the abnormal phenotype of ZS-1TN1812 was due to the overexpression of *CmSIT1*. Therefore, an overexpression vector pOESIT1 was constructed and transformed into the wild-type strain ZS-1 using the *Agrobacterium*-mediated transformation system as described by Li et al. (2005). Primer SIT1-SP (OVER-T)/SIT1-AP(OVER-T) were used to obtain the full length cDNA of *CmSIT1*.

### Determination of *CmSIT1* expression in *C. minitans* in contact with host fungus and non-host fungus

To determine whether *CmSIT1* could be expressed when *C. minitans* is contacting its host or non-host fungi, strain ZS-1 of *C. minitans*, strain Ep-1PNA367 of *S. sclerotiorum*, and strain Ch-1 of *C. higginsianum* were grown on CM-PDA plates for 3 days. The mycelial mass (about 0.25 g) of *C. minitans* was obtained and mixed with either an equal mycelial mass of strain Ep-1PNA367 or strain Ch-1 in a sterilized mortar. The mixed mycelial mass was amended with 1 μL ddH_2_O, and then ground finely with a pestle. About 200 μL homogenate was sampled and spread on CM-PDA plates and allowed to grow at 20°C for 2, 4, or 5 days. The mycelial mass of the culture mixture was sampled to extract RNA for determining the transcripts of C*mSIT1* with quantitative real time PCR (qRT-PCR). The first strand cDNA was reverse-transcribed with the kit described above and used for quantitative PCR (qPCR) analysis with the CFX96 Real-Time PCR Detection System (Bio-Rad, USA). The reaction volume was 20 μL and each sample had three replicates. The program was as follows: denaturation at 95°C for 1 min, followed by 49 amplification cycles of 95°C for 10 s and 58°C for 30 s. The melt curve was generated to verify the specificity of the amplification (from 65 to 95°C with an increment of 0.5°C per cycle, with each cycle held for 5 s). The mycelial mass of strain ZS-1 was ground and spread on cellophane membrane laid on PDA and grown 2, 4, or 5 days and then harvested for RNA extraction and qRT-PCR analysis as controls, and the expression of *C. minitans actin* was used as a reference gene. The primer sequences QPCR-FP1/ QPCR-RP1 and CmACT289/CmACT419 were listed in Table 1. This experiment was performed four times.

### Iron stress assay

To test the fungal strain for iron stress tolerance, different concentrations of ferric chloride (1, 1.5, 2, or 2.5 mM) were added in 20 mL PDA in 90 mm Petri dishes. An activating hyphal agar plug of *C. minitans* was inoculated at the center of each plate. The plates were cultured at 20°C in an incubator for 12 days. The PDA plates, without any emendation, were used as controls. After 12 days, photographs of colonies were taken and colony diameters were measured. This experiment was repeated four times.

### Iron level determination in *C. minitans*

To determine the possible accumulation of iron in mutant and wild-type colonies of *C. minitans*, iron concentrations in hyphal masses were measured. Both the mutant ZS-1TN1812 and the wild-type strain ZS-1 were grown on CM-PDA plates for 2 days, and then the mycelial mass was harvested for iron determination. For each sample, the 100 mg mycelial mass was finely ground under liquid nitrogen with a mortar and pestle. The hyphal powder was transferred to a clean 5 mL-tube with 2 mL ice-cold PBS buffer. The mixture was further subjected to sonication with an ultrasonic cleaner (SB5200DT, Wuhan, China) for 20 min, on ice-water. Then, the liquid in the 5 mL-tube was centrifuged at 12,000 rpm for 10 min at 4°C. The clear supernatant was transferred into a clean 2 mL-tube. The supernatant samples were either stored at −80°C or subjected to iron concentration determination. The Quantichrom™ Iron Assay Kit (Bioassay Systems, USA) was used to determine the iron concentration, following the manufacturer's instructions. The iron concentration was measured at a wavelength of 590 nm with an automatic micro plate reader (BOX 998, BioTek® Instruments, Inc. USA). PBS was used instead of a sample as the blank control. This experiment was performed 3 times.

### Data analyses

SAS version 8.1 (SAS Institute, Inc., Cary, NC, USA) was used to analyze the variation between treatments for each experiment using an ANOVA. When significant treatment effects were found, means of different treatments were compared using the protected least significant difference test at *P* = 0.05.

## Results

### ZS-1TN1812 has abnormal phenotypes

**Figure 1:**
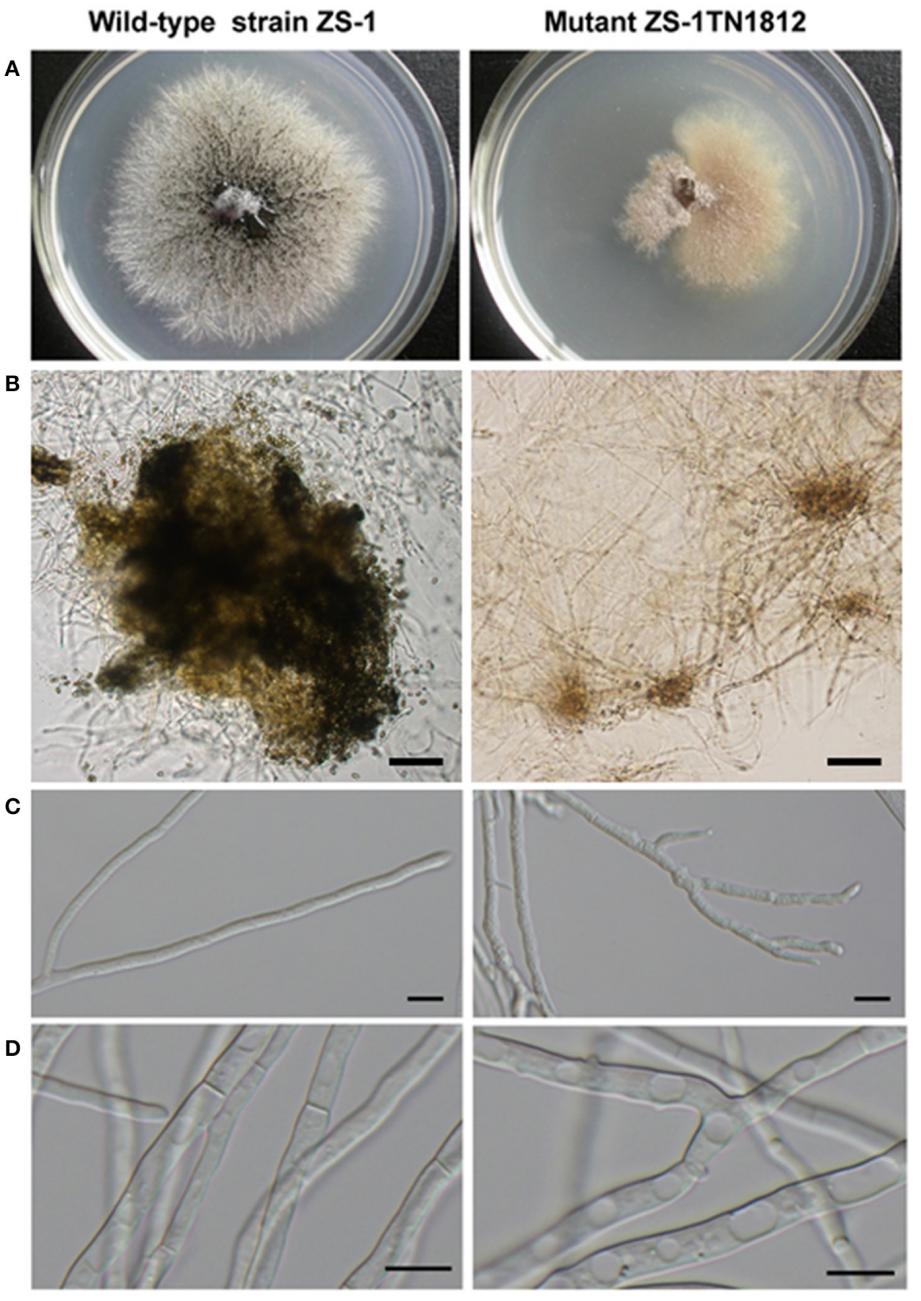
Comparison of the colony morphology and hypha between the *C. minitans* strain ZS-1TN1812 and ZS-1. **(A)** Colony morphology, mutant ZS-1TN1812 and the wild-type strain ZS-1 grew on PDA medium for 11 days at 20–22°C; **(B)** ZS-1TN1812 produced a few unmature pycnidia without conidia, while strain ZS-1 formed a great deal of mature pycnidia and conidia; Bar = 100 μm. **(C,D)** Hypha and hyphal tips. Hypha and hyphal tips were observed after inoculating 3 days under light microscope (Nikon, Japan); Bar = 10 μm.

ZS-1TN1812 grew slowly in sectors on PDA plates, with the growth rate being about 1.4 mm/d, while the wild-type strain, ZS-1, was about 3.0 mm/d. The hyphal tips of mutant ZS-1TN1812 branched excessively, and the hyphae in the colony centers were frequently vacuolated. The mutant, ZS-1TN1812, may have produced a few pycnidia, but no conidia were formed in the pycnidia, while 1.3 × 10^9^ conidia were produced in each 9 cm Petri dish by strain ZS-1 14 days post-incubation. The colony of the mutant strain developed on PDA, with a yellow to brown appearance, while the typical colony of the wild-type strain, ZS-1 was filled with dark pycnidia (Figure 1). Furthermore, mutant ZS-1TN1812 could not parasitize and cause the decay of *S. sclerotiorum* sclerotia after 30 days, while strain ZS-1 decayed the sclerotia completely.

### ZS-1TN1812 could produce many antifungal substances

**Figure 2:**
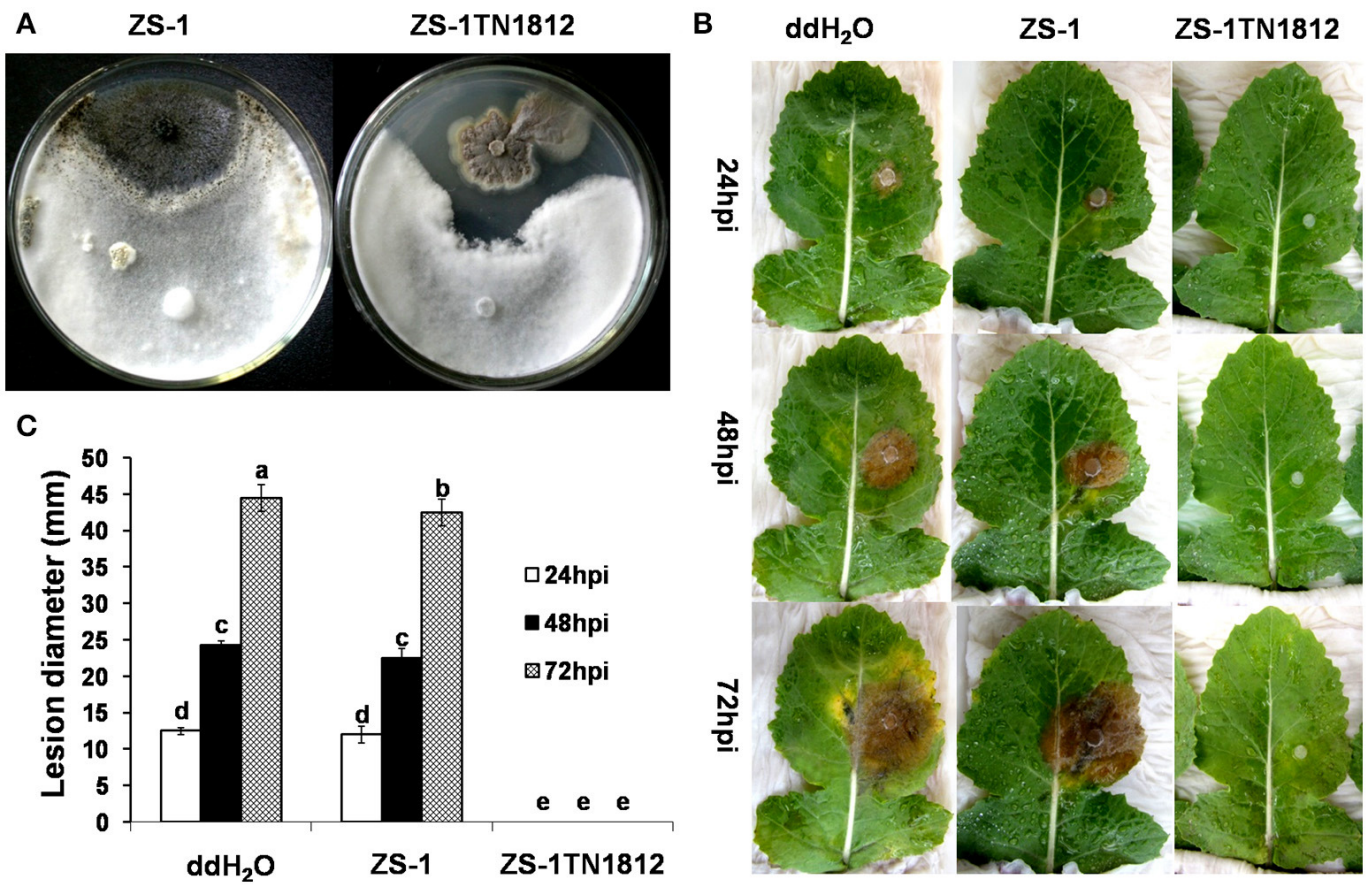
*C. minitans* mutant ZS-1TN1812 strongly inhibited the growth and infection of *S. sclerotiorum* both on PDA and on rapeseed leaves. **(A)** ZS-1TN1812 dual-cultured with *S. sclerotiorum*, showing an inhibition zone. The wild-type strain ZS-1 was used as a control. Co-inoculated plates were placed at 20°C for 7 days. **(B,C)** The filtrate of ZS-1TN1812 fully suppressed the infection of *S. sclerotiorum* on rapeseed leaves. The hyphae were removed by passing through three layers of filter paper and possible hyphal debris was removed by passing through a bacterial filter (0.22 μm). Rapeseed leaves were sprayed with the filtrate until fully wet. Bars indicate standard error. Error bars indicate the SD from three replicate means. Means followed by the different letters on the top of each column are significantly different at the *P* < 0.05 level of confidence according to Duncan's multiple range test.

*C. minitans* is able to produce antifungal substances (AFS) under low pH conditions, but this ability is weakened when growing on PDA medium (McQuilken et al., 2002; Yang et al., 2008). However, ZS-1TN1812 could produce a large amount of antifungal substances. When dual-cultured with strain Ep-1PNA367 on PDA, ZS-1TN1812 inhibited the growth of *S. sclerotiorum*, the two colonies did not intermingle with each other, and the inhibition zone was maintained indefinitely. While the wild-type strain ZS-1 colonies intermingled with the colonies of *S. sclerotiorum*, no inhibition zone could be observed. Similar inhibition was observed when dual-culturing on low-pH MCD medium (data not shown). Furthermore, the filtrate of ZS-1TN1812 could suppress the infection of *S. sclerotiorum* on rapeseed leaves, while the filtrate of the wild-type strain ZS-1 could only slightly delay the infection of *S. sclerotiorum* (Figure 2).

*C. minitans'* AFS may contain different components. We detected the antagonistic ability of AFS against *S. sclerotiorum* (host) and *C. higginsianum* (non-host). When 2 mL filtrate of ZS-1TN1812 was amended into 18 mL PDA medium, the filtrate almost fully inhibited the hyphal growth of *S. sclerotiorum* and *C. higginsianum*. However, when the filtrate was treated with high temperature or proteinase K, the antagonistic ability of the filtrate to *S. sclerotiorum* and *C. higginsianum* was significantly different. The antagonistic ability of 100°C-treated filtrate to *S. sclerotiorum* was not significantly different from non-treated filtrate, but the antagonistic ability was fully lost when the filtrate was treated with 120°C heat. The antagonistic ability was also maintained when the filtrate was treated with proteinase K. These data suggested that the antifungal component against *S. sclerotiorum* is not likely to be a protein.

**Figure 3:**
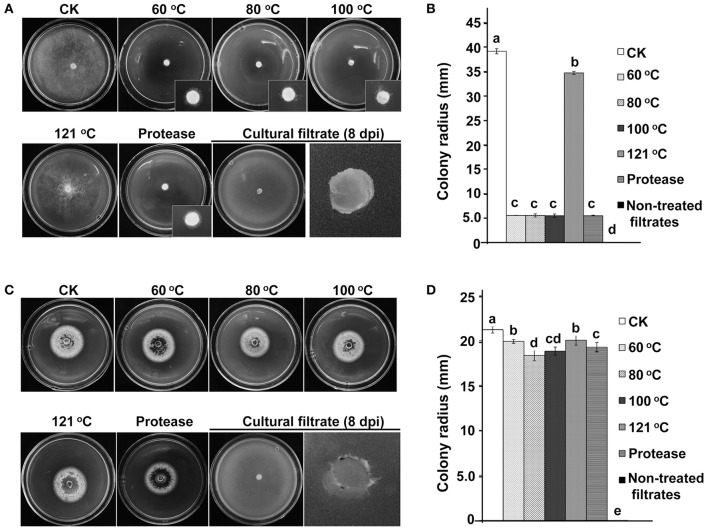
Two kinds of antifungal substances produced by the *C. minitans* mutant ZS-1TN1812. **(A,B)** Antifungal substances that inhibit *S. sclerotiorum* were thermo-tolerant and protease-tolerant, suggesting the antifungal substances were not proteinous substances. **(C,D)** Antifungal substances that inhibit *C. higginsianum* were thermo-sensitive and protease-sensitive, suggesting that the antifungal substances to *C. higginsianum* were proteinous substances. Non-treated filtrates of ZS-1TN1812 fully suppressed the hyphal growth of both *S. sclerotiorum* and *C. higginsianum*. Bars indicate standard error. Means followed by the different letters on the top of each column are significantly different at the *P* < 0.05 level of confidence according to Duncan's multiple range test.

Interestingly, although the non-treated filtrate produced by ZS-1TN1812 could inhibit the growth of *C. higginsianum*, the filtrate completely lost the activity when bathed at 60°C or treated with proteinase K (Figure 3). The phenomenon suggested that the antifungal component against *C. higginsianum* is very likely to be a proteinous substance. Thus, the antifungal substances produced by ZS-1TN1812 may contain both a proteinous component and a non-proteinous component, with *S. sclerotiorum* being sensitive to the non-proteinous substances, while *C. higginsianum* is sensitive to the proteinous substance.

### T-DNA insertion activated the expression of *CmSIT1* in ZS-1TN1812

**Figure 4:**
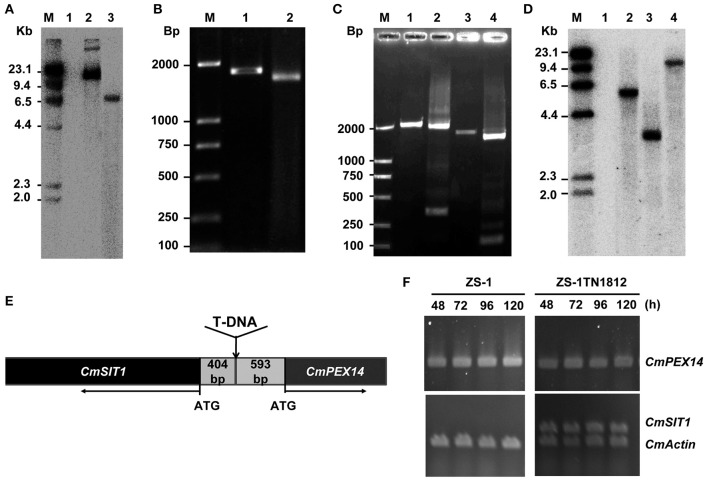
T-DNA insertion and the disrupted gene in *C. minitans* mutant ZS-1TN1812. **(A)** Southern blot analysis of the copy number of T-DNA; the genomic DNA of ZS-1TN1812 and the wild-type strain ZS-1 were digested with *Sac*I. Lane M, DNA weight marker, λ DNA digested with *Hin*d?; Lane 1, ZS-1; Lane 2, Plasmid DNA used for transformation; Lane 3, the mutant ZS-1TN1812. Hygromycin resistance gene (*HPH*) labeled with α-^32^P was used as the probe. **(B)** Amplification of the region flanking the left side of T-DNA in ZS-1TN1812 using iPCR; Lane 1 and lane 2 were the PCR products of the first round and the second round amplification. **(C)** The full length *CmSIT1* DNA sequences obtained by utilizing iPCR; Lane 1-2, iPCR products of the left border genome DNA; Lane 3–4, iPCR products of the region flanking the right side of T-DNA; **(D)** *CmSIT1* has only one copy in the genome of *C. minitans* as determined with Southern blot analysis. Lane M, DNA weight marker, λ DNA digested with *Hin*d?; Lane 1, Mock; Lane 2-4, genomic DNA digested by *Eco*RI, *Pst*I, and *Xba*I. **(E)** Diagram of T-DNA insertion site, T-DNA was inserted at the non-transcription region between two putative genes, *CmSIT1* and *CmPEX14*. **(F)** RT-PCR analysis of the expression pattern of putative *CmSIT1* and *CmPEX14* in the wild-type strain ZS-1 and mutant ZS-1TN1812 cultured on PDA medium.

Southern blot analysis showed only one T-DNA insertion in ZS-1TN1812 (Figure 4A). A DNA segment of ~1,800 bp was successfully amplified with inverse PCR from the region flanking the left side of T-DNA, after the genomic DNA of ZS-1TN1812 was digested with *Kpn* I (Figure 4B). The DNA fragment obtained was sequenced and used to search a local *C. minitans* ZS-1 genome database. The right flank of the inserted T-DNA was confirmed with PCR amplification (Figure 4C). We found that there were two putative genes flanking the T-DNA insertion. These two genes were arranged in the opposite direction and the un-translation space between the two genes was 997 bp (Figure 4E). Flanking the right side of the T-DNA insertion was a putative gene encoding a protein that is a homolog of peroxisomal membrane anchor protein 14 (PEX14), and flanking the left side was a putative gene encoding a protein that is a homolog of the siderochrome iron transporter (SIT). The two putative genes were named *CmPEX14* and *CmSIT1*, respectively.

To probe if these two genes were expressed in the wild-type and mutant strains, the total RNA was extracted from the mycelial mass collected from colonies growing for 2 to 5 days on PDA plates and RNA samples were examined with RT-PCR amplification. The results showed that *CmPEX14* was constitutively expressed in both the wild-type strain and in ZS-1TN1812, suggesting that the T-DNA insertion in the mutant did not significantly destroy *CmPEX14* (Figure 4F). Surprisingly, the expression of *CmSIT1* in the wild-type strain was not detected from these RNA samples, but was constitutively detected in ZS-1TN1812 (Figure 4F). Thus, the *CmSIT1* was activated by insertion of T-DNA, and we supposed that the abnormal phenotype of mutant was possibly due to the overexpression of *CmSIT1*.

### *CmSIT1* encodes a putative siderochrome-iron transporter

**Figure 5:**
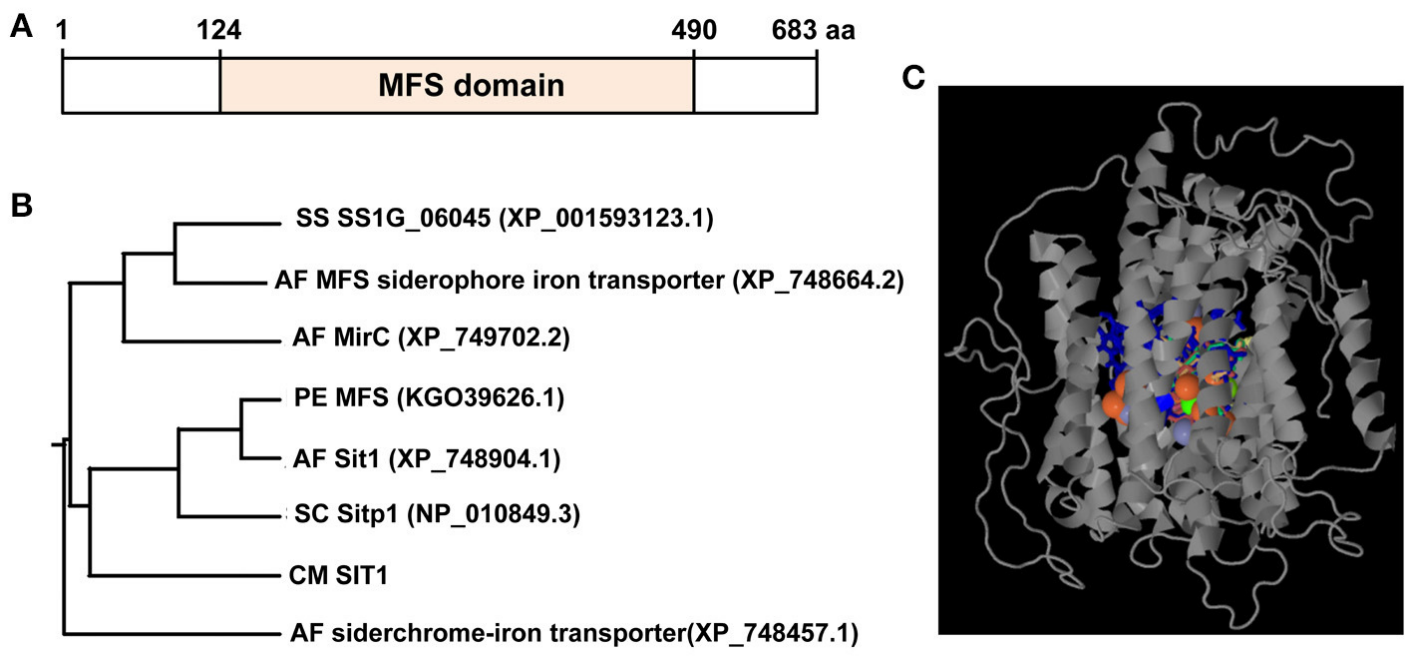
*C. minitans* CmSIT1 has a conserved domain (aa124-aa490) of the major facilitator superfamily (MFS) transporter. **(A)** A diagram of CmSIT1 predicted with the Blastp program from the NCBI website; **(B)** A phylogenetic tree was constructed, using the MEGA5 program, based on reference siderophore transporter proteins from *A. fumigatus, P. expansum, S. cerevisiae*, and *S. sclerotiorum*. **(C)** The three dimensional (3D) model of CmSIT1 protein, which was generated with the Phyre2 program. The binding site was presented with colored amino acidic residues.

The full length *CmSIT1* is 2,510 bp, with seven exons and the full length of cDNA is 2,052 bp, coding for a putative protein with 683 amino acid residues (GenBank Acc. No. MF447899). Southern blot analysis showed only one copy of *CmSIT1* in the genome of *C. minitans* (Figure 4D). This putative protein may have 13 transmembrane domains, as predicted with TMHMM Server v. 2.0 (http://www.cbs.dtu.dk/services/TMHMM/). Blastp analysis using the NCBI database showed that CmSIT1 had a conserved domain (amino acid 124–amino acid 490) of the MFS transporter (cd06174) (Figure 5A). This conserved domain was also identified with the Phyre2 and RaptorX programs, showing that the heterogens in the predicted binding site contained metal ions and most are iron ions. A structural model of CmSIT1 was constructed with the *E. coli* multidrug transporter MdfA1pw4:A (Heng et al., 2015) as a template and the iron-binding site was displayed (Figure 5B). Thus, CmSIT1 is most likely a MFS transporter for iron uptake.

Siderophore-mediated Fe^3+^ uptake in fungi is accomplished through SIT transporters, a subfamily of the major facilitator superfamily. Blastp analysis showed that CmSIT1 has homologs with other fungi and it was highly homologous to the MFS transporter of *Penicillium expansum* (KGO39626.1), with 31% (183/585) identity, 54% similarity (319/585), and a 2% (17/585) gap. CmSIT1 also has considerable identity (25%, 141/569) and similarity (48%, 274/569) to the SIT1 protein of the *S. cerevisiae* strain S288c (NP_010849.3). The identity and similarity of CmSIT1 and other selected proteins were listed in Table 2. A phylogenetic tree analysis was constructed based on reference proteins from *A. fumigatus, P. expansum, S. cerevisiae*, and *S. sclerotiorum* (Figure 5C), and CmSIT1 was clustered with Sitp1 of *S. cerevisiae*, Sit1 of *A. fumigates* and MFS, a general substrate transporter of *P. expansum*. This further suggested that CmSIT1 is a SIT transporter.

**Table 2:** The identities and positives between CmSIT1 of *C. minitans* and selected fungal homologs.

**Fungal namue**	**Gene name or function**	**Accession no**.	**Score**	**Expect**	**Identities**	**Positives**	**Gaps**	**References**
*Penicillium expansum*	Major facilitator superfamily domain, general substrate transporter	KGO39626.1	298 bits (764)	7e-88	183/585 (31%)	319/585 (54%)	17/585 (2%)	Ballester et al., 2015
*Fonsecaea pedrosoi* CBS 271.37	hypothetical protein Z517_06750	XP_013283943.1	298 bits (764)	9e-88	180/571 (32%)	303/571 (53%)	24/571 (4%)	Cuomo et al., 2015, unpublished
*Penicillium roqueforti* FM164	Siderophore iron transporter 1	CDM27023.1	298 bits (763)	1e-87	178/559 (32%)	313/559 (55%)	12/559 (2%)	Cheeseman et al., 2014
*Penicillium digitatum* Pd1	hypothetical protein PDIP_84890	XP_014532645.1	297 bits (761)	2e-87	179/581 (31%)	316/581 (54%)	18/581 (3%)	Marcet-Houben et al., 2012
*Coccidioides immitis* RMSCC 2394	siderophore iron transporter 1	KMP06227.1	297 bits (760)	4e-87	174/562 (31%)	301/562 (53%)	18/562 (3%)	Henn et al., 2015 unpublished
*Rhinocladiella mackenziei* CBS 650.93	hypothetical protein Z518_07380	XP_013270963.1	295 bits (756)	1e-86	181/607 (30%)	313/607 (51%)	28/607 (4%)	Cuomo et al., 2015, unpublished
*Exophiala aquamarina* CBS 119918	MFS transporter, SIT family, siderophore-iron:H+ symporter	XP_013254158.1	296 bits (757)	1e-86	186/599 (31%)	310/599 (51%)	27/599 (4%)	Cuomo et al., 2015, unpublished
*Coniosporium apollinis* CBS 100218	hypothetical protein W97_06828	XP_007783002.1	295 bits (754)	2e-86	172/556 (31%)	301/556 (54%)	10/556 (1%)	Cuomo et al., 2014, unpublished
*Coccidioides immitis* RS	siderochrome-iron transporter Sit1	XP_001240692.2	294 bits (752)	5e-86	174/558 (31%)	301/558 (53%)	14/558 (2%)	Neafsey et al., 2015, unpublished
*Coccidioides posadasii* C735 delta SOWgp	Major Facilitator Superfamily protein	XP_003067856.1	293 bits (751)	7e-86	175/558 (31%)	301/558 (53%)	14/558 (2%)	Sharpton et al., 2009
*Colletotrichum orbiculare* MAFF 240422	siderophore iron transporter 1	ENH80818.1	293 bits (751)	9e-86	166/548 (30%)	292/548 (53%)	13/548 (2%)	Gan et al., 2015, unpublished
*Histoplasma capsulatum* NAm1	Conserved hypothetical protein	XP_001536364.1	293 bits (749)	1e-85	168/541 (31%)	289/541 (53%)	10/541 (1%)	Birren et al., unpublished
*Penicillium italicum*	Major facilitator superfamily domain, general substrate transporter	KGO73837.1	292 bits (748)	1e-85	177/571 (31%)	312/571 (54%)	21/571 (3%)	Ballester et al., 2015
*Emmonsia crescens* UAMH 3008	MFS transporter, SIT family, siderophore-iron:H+ symporter	KKZ63768.1	290 bits (743)	8e-85	169/553 (31%)	289/553 (52%)	24/553 (4%	Cuomo et al., 2014, unpublished
*Penicillium solitum*	Major facilitator superfamily	KJJ23129.1	289 bits (739)	1e-83	178/606 (29%)	321/606 (52%)	19/606 (3%)	Yu et al., 2015, unpublished
*Penicillium rubens* Wisconsin 54-1255	Pc13g04290	XP_002558865.1	287 bits (734)	1e-83	174/581 (30%)	316/581 (54%)	18/581 (3%)	van den Berg et al., 2008
*Colletotrichum higginsianum*	major facilitator superfamily transporter	CCF35502.1	286 bits (733)	3e-83	183/598 (31%)	305/598 (51%)	36/598 (6%)	Ma et al., 2102, unpublished
*Colletotrichum fioriniae* PJ7	major facilitator superfamily transporter	XP_007590024.1	286 bits (733)	4e-83	178/617 (29%)	318/617 (51%)	31/617 (5%)	Baroncelli et al., 2014

### Overexpression of *CmSIT1* was responsible for the abnormal phenotype of ZS-1TN1812

**Figure 6:**
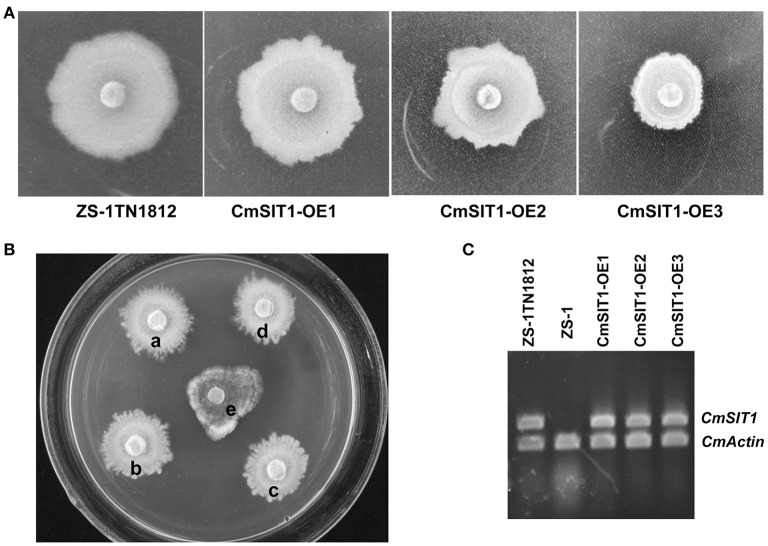
The colony morphology of the CmSIT1-overexpression transformants of *C. minitans*. **(A)** Colony morphology of mutant ZS-1TN1812 and three over-expression transformants which cultured on PDA medium at 20°C for 10 days. **(B)** Transformants also have strong antagonistic ability, (a) ZS-1TN1812, (b) CmSIT1-OE1, (c) CmSIT1-OE2, (d) CmSIT1-OE3, and (e) Ep-1PNA367. *S. sclerotiorum* stain Ep-1PNA367 was inoculated after ZS-1TN1812 and transformants growing on PDA for 4 days. Then all the strains were co-cultured for 7 days at 20°C. **(C)** RT-PCR analysis the expression of *CmSIT1* gene in CmSIT1-overexpression transformants. Strains were cultured on PDA medium for 48, 72, 96, or 120 h, and then the mycelia were mixed together equally to extract the total RNA.

The full length cDNA of *CmSIT1* was cloned into the pCXH vector, transformed into *A. tumefaciens* EHA105, and then transformed into the wild-type strain ZS-1. The phenotypes, including growth rate, colony morphology, and production of antifungal substances, of the over-expression transformants were similar to that of ZS-1TN1812. The colony morphologies of over-expression transformants were shown in Figure 6A and the growth suppression of *S. sclerotiorum* was shown in Figure 6B. The *CmSIT1* expression of overexpression transformants was also confirmed with RT-PCR (Figure 6C). Thus, the consistent expression of *CmSIT1* could cause abnormal growth and excessive synthesis of antifungal substances.

### High accumulation of iron in ZS-1TN1812

**Figure 7:**
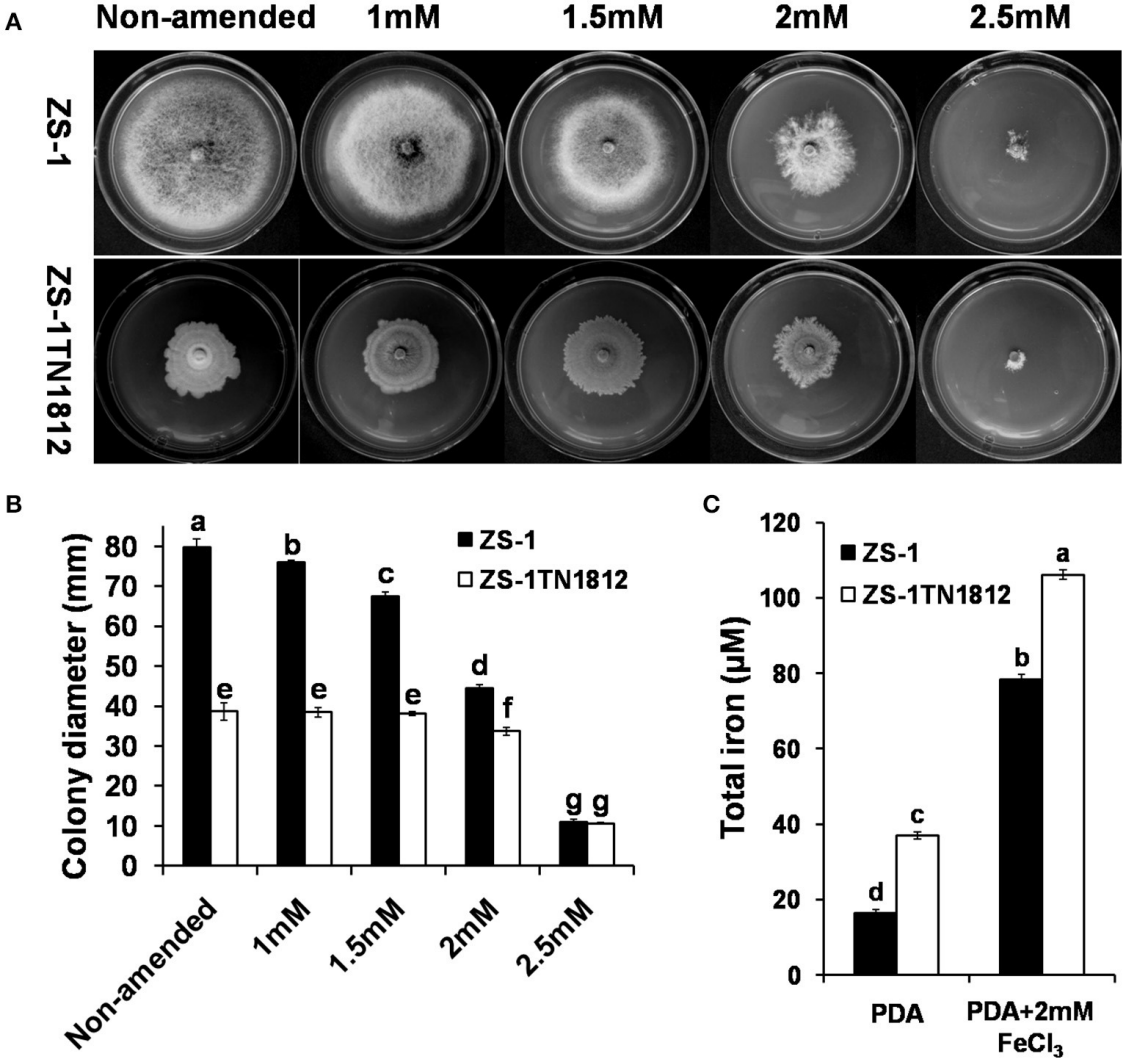
*C. minitans* strains grew on ferric chloride (FeCl_3_)-amended PDA. **(A,B)** Colonies of ZS-1TN1812 and the wild-type strain ZS-1 of *C. minitans* developed on media after 12 days, PDA without any emendation were used as controls. **(C)** Determination of iron content in hypha of strains ZS-1TN1812 and ZS-1. Bars indicate standard error. Error bars indicate the SD from four replicate means for **(B)** and three for **(C)**. Means followed by the different letters on the top of each column are significantly different at the *P* < 0.05 level of confidence according to Duncan's multiple range test.

Iron homeostasis is very important for most organisms. We suspected that the abnormal phenotype of ZS-1TN1812 is likely to be caused by excess uptake of the iron element since *CmSIT1* encodes a putative siderophore iron transporter. To test this hypothesis, both the wild-type and mutant strains were grown on PDA medium amended with different concentrations of ferric chloride and the results showed that the growth of the wild-type strain was inhibited when growing on 1 mM FeCl_3_-amended PDA medium. When the concentration of FeCl_3_ was increased, the inhibition was more significant, with wild-type strain growth almost completely inhibited when the concentration of FeCl_3_ in medium was 2.5 mM. The growth of ZS-1TN1812 in 2 mM FeCl_3_-amended PDA medium was not significantly different from that of non-amended PDA medium. However, complete inhibition of growth was observed when growing on 2.5 mM FeCl_3_-amended PDA medium. Interestingly, the colony morphology of the wild-type strain developed on 2 mM FeCl_3_-amended PDA medium was similar to that of ZS-1TN1812 on PDA medium (Figures 7A,B).

As an essential element, it is not surprising that iron could be detected in the hyphal mass of strains ZS-1 and ZS-1TN1812. However, the iron level in ZS-1TN1812 was significantly higher than in strain ZS-1 when growing on the PDA plate, with the iron concentration at 37.2 μM in the mutant and only 16.3 μM in the wild-type strain ZS-1. When growing on 2.0 mM FeCl_3_-amended PDA, the iron levels in strain ZS-1 and strain ZS-1TN1812 were significantly higher, at 78.5 and 106.2 μM (Figure 7C), respectively. This result combined with the results above suggested that iron-uptake ability in ZS-1TN1812 was significantly increased.

### *CmSIT1* is expressed when *C. minitans* contacts either host or non-host fungi

**Figure 8:**
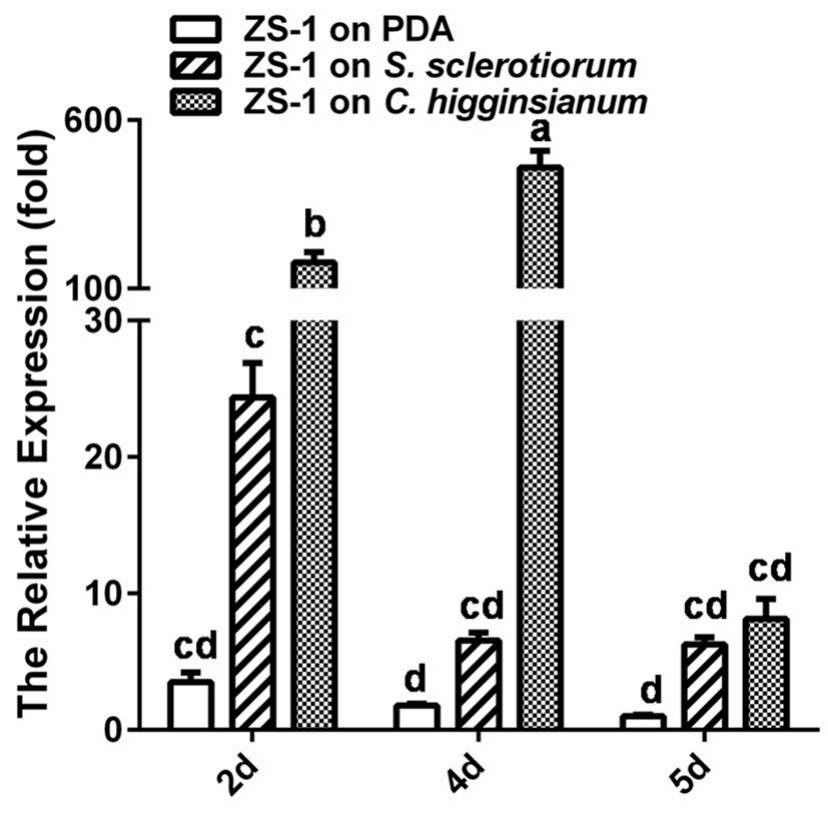
Inducible expression of *CmSIT1* in *C. miniatns* when contacting with its host, *S. sclerotiorum* and non-host fungus, *C. higginsianum* 2, 4, and 5 dpi. The relative levels of transcript were calculated by the comparative Ct method. The levels of *actin* transcript were used to normalize different samples. The relative expression level of *CmSIT1* in the wide-type stain ZS-1 for 5 d was assigned as value 1. Bars represent means and standard deviations (three replications). Error bars indicate the SD from four replicate means. Means followed by the different letters on the top of each column are significantly different at the *P* < 0.05 level of confidence according to Duncan's multiple range test.

Although, the expression of *CmSIT1* is not detectable via RT-PCR when growing on PDA medium alone, it may be expressed when dual-cultured with other microorganisms, since siderophores are important molecules in competition with other organisms for iron. To test this hypothesis, *C. minitans* was allowed to contact its host, *S. sclerotiorum*, or non-host, *C. higginsianum*, and the expression of CmSIT1 was determined using qRT-PCR. The results showed that when contacting *S. sclerotiorum, CmSIT1* was significantly expressed at 2 days post-inoculation (dpi), and the expression declined at 4 and 5 dpi, but the expression level was still significantly higher than when growing on PDA alone. When *C. minitans* and non-host *C. higginsianum* grew on PDA, *CmSIT1* was highly expressed at 2 dpi and the high level of expression was maintained even at 4 dpi, but dropped significantly at 5 dpi (Figure 8). This result suggested that *CmSIT1* may play a role during parasitizing, such as competing for iron with its host, *S. sclerotiorum*.

## Discussion

Our research identified a siderophore iron transporter gene *CmSIT1* from the sclerotial parasite *C. minitans*. CmSIT1 has 683 amino acid residues with a conserved domain (amino acid 124–490) of the MFS transporter. CmSIT1 is relatively large compared to all other fungal SIT1 homologs. Some other large SIT proteins are the MFS transporter (KJJ23129.1) of *Penicillium solitum*, with 651 aa, and a hysipothetical protein CPAR2-803030 (CCE41752.1) of *Candida parapsilosis*, with 648 aa, and a putative siderophore iron transporter, mirb (GAP91069.1), from *Rosellinia necatrix*, with 647 aa. An annotated hypothetical protein (XP_001593123.1) and homolog of *CmSIT1* was identified from *S. sclerotiorum*, the host of *C. minitans*. The expression of *CmSIT1* was not detected by RT-PCR when growing on PDA medium, while it could be detected when dual-cultured with its host. Overexpression of *CmSIT1* in *C. minitans* led to extremely slow growth on PDA medium and development of an abnormal colony morphology. Importantly, it could produce large levels of antifungal substances to antagonize the growth of *S. sclerotiorum*, and when sprayed on leaves of rapeseed, it could inhibit the lesion formation induced by *S. sclerotiorum*.

*C. minitans* produces AFS, specifically suppressing *S. sclerotiorum*. McQuilken et al. (2002) found that *C. minitans* could produce antifungal metabolites and the production of these could be affected by nutritional factors. After that, they identified four closely related metabolites from filtrates of *C. minitans*, one of which was macrosphelide A. Macrosphelide A had a wide antagonistic spectrum (McQuilken et al., 2003; Tomprefa et al., 2009). Yang et al. (2007) reported that the filtrate of *C. minitans* had antifungal activity and could suppress the germination of *S. sclerotiorum* ascospores and inhibit the expansion of lesions induced by *S. sclerotiorum* on rapeseed leaves, and there was a possibility that the antifungal activity in the filtrate may partially contribute to the fungal-cell wall enzymes of *C. minitans*. Yang et al. (2008) found that both ambient pH and nutritional factors affected the antifungal activity of the mycoparasite *C. minitans* and this finding led to the recent discovery that CmpacC regulates mycoparasitism, oxalate degradation, and antifungal activity of *C. minitans* (Zeng et al., 2014; Lou et al., 2015). Interestingly, the PacC-mediated ambient-pH regulatory system in *A. nidulans* was found to regulate the biosynthesis and uptake of siderophores (Eisendle et al., 2004). Our findings suggested that *C. minitans* could produce antifungal substances specifically inhibiting the growth of *S. sclerotiorum* and that CmSIT1 was involved in the production of these antifungal substances. In the wild-type strain, *CmSIT1* was only highly expressed at the early stages of contact with its host, *S. sclerotiorum*. This finding suggested that the antifungal substances may transiently function at the early stages of the interaction between *C. minitans* and *S. sclerotiorum. C. minitans* could regulate the balance between production of antifungal substances and mycoparasitism, since *C. minitans* is not capable of completely suppressing its host's growth on rapeseed, as was our original expectation.

Our study suggested that *C. minitans* had the potential to produce several kinds of antagonistic substances. In ZS-1TN1812, the antifungal substances could be classified into two groups based on thermo-tolerance, namely non-thermo-tolerant antifungal substances, and thermo-tolerant antifungal substances. Because the thermo-tolerant antifungal substances could specifically suppress the growth of *S. sclerotiorum*, this antifungal substance was not likely to be macrosphelide A, which has a wide antagonistic spectrum. Of striking contrast with the wild-type strain, spraying the filtrate of ZS-1TN1812 on the leaves of rapeseed could highly inhibit the infection and lesion formation of *S. sclerotiorum*, suggesting that the antifungal substances produced by this mutant could be effective on the leaves. Further investigation into the substances produced by ZS-1TN1812, such as isolation, identification, and characterization, may lead to the discovery of new chemicals against plant diseases caused by *S. sclerotiorum*.

Indeed, *C. minitans* has to overcome competition and threat from microorganisms other than parasitizing *S. sclerotiorum*. We found that ZS-1TN1812 could produce a proteinous antifungal substances that could significantly inhibit the growth of *C. higginsianum*, and found that the expression of *CmSIT1* in the wild-type strain of *C. minitans* could be stimulated when growing on PDA with *C. higginsianum*, suggesting that *C. minitans* could explore the siderophore transporter system by taking in iron from ambient environments, and this may lead to competition with other organisms for iron and stimulate the synthesis of aproteinous antifungal substances that work against other fungi. Thus, *CmSIT1* is likely to play an important ecological role for *C. minitans*, allowing it to survive in nature.

In *Acremonium chrysogenum*, a producer of the beta-lactam antibiotic cephalosporin C, starvation could induce the production of the extracellular siderophores and the expression of a putative siderophore transporter gene, mir1 (Gsaller et al., 2012). We currently have not investigated if *C. minitans* produces siderophores or if these siderophores could be synthesized and secreted under *CmSIT1* expression. However, we found that the colony morphology of the wild-type strain, when grown on 2 mM FeCl_3_-amending PDA medium, was similar to that of ZS-1TN1812, suggesting the abnormal phenotype of ZS-1TN1812 was possibly due to high iron accumulation of in fungal cells. This hypothesis was confirmed by the detection of iron in mycelium of strain ZS-1TN1812 and ZS-1 when growing on PDA and 2 mM FeCl_3_-amended PDA medium.

Siderophores are considered one of the mechanisms through which some beneficial microorganisms antagonize plant pathogens (Berendsen et al., 2015). Fungi may produce several kinds of siderophores, with the exception of *S. cerevisiae*, which does not synthesize any siderophore, and siderophore-iron transporters may only specifically bind and transport certain types of siderophores. Although only one putative siderophore iron transporter, SS1G_06045 (XP_001593123.1), could be found with the Sitp1 of *S. cerevisiae* as the seed sequence, SS1G_06045 is phylogenetically distant from CmSIT1, suggesting that *C. minitans* and its host, *S. sclerortiorum*, may use different siderophores to bind iron and *C. minitans* may weaken the resistance of *S. sclerotiorum* to establish mycoparasitism at the early stages of infection.

*CmSIT1* is a potential gene for improving the control ability of *C. minitans*. Although, *C. minitans* could produce antifungal substances, the amount of antifungal substances produced by the wild-type strain is not enough to suppress the growth of *S. sclerotiorum*. Usually a no-inhibition zone could be observed when dual-culturing on PDA medium and *C. minitans* could not efficiently suppress the lesion expansion induced by *S. sclerotiorum* on rapeseed leaves. This is disadvantageous and limits the widespread use of *C. minitans*, leading to difficulty in aerial parts control, which contributes to Sclerotinia disease. In our study, we found that the antifungal substances produced by ZS-1TN1812 were likely to contain two types of active compounds, namely, non-proteinous antibiotics, and proteinous antibiotics. *S. sclerotiorum* showed more tolerant to the proteinous antibiotics produced by the mutant and was specifically sensitive to the non-proteinous antifungal substances, while another fungus, *C. higginsianum*, was sensitive to the proteinous antibiotics. Overexpression of *CmSIT1* proved to be a double-edged sword, as it improved the production of antifungal substances, while it also restricted the growth, conidiation, and parasitization of *C. minitans*. Tightly controlled expression of *CmSIT1* through genetic modification may strengthen the antagonistic ability of *C. minitans* during interaction with *S. sclerotiorum*.

## Conclusion

In this research, *CmSIT1*, a gene involved in siderophore-mediated iron transport, was cloned and the functions were studied in *C. minitans*, a mycoparasite of *S. sclerotiorum*. Expression of *CmSIT1* led to reduced growth and enhanced antifungal ability. The antifungal substances could significantly inhibit the infection of *S. sclerotiorum* on the leaves of rapeseed.

## Author contributions

XS, DJ, and YF designed the research and wrote the paper; XS, YZ, JJ, JX, and JC executed the experiments. JC, JX, YZ, HL, and YF performed the data and bioinformatics analyses. All authors read and approved the final manuscript.

### Conflict of interest statement

The authors declare that the research was conducted in the absence of any commercial or financial relationships that could be construed as a potential conflict of interest. The reviewer JM-A and handling Editor declared their shared affiliation.
